# *Echinostoma malayanum* Infection, the Philippines

**DOI:** 10.3201/eid1307.061486

**Published:** 2007-07

**Authors:** Vicente Y. Belizario, Giovanni G. Geronilla, Marilyn Benedith M. Anastacio, Winifreda U. de Leon, Adriano P. Suba-an, Arlene C. Sebastian, Michael J. Bangs

**Affiliations:** * National Institutes of Health, Manila, the Philippines; † University of the Philippines Manila, Manila, the Philippines; ‡ Health Development, Caraga Region, Butuan City, the Philippines; § Rural Health Unit, Santa Monica, the Philippines; ¶ Navy Region Northwest, Silverdale, Washington, USA

**Keywords:** intestinal parasitoses, heterophyid, *Echinostoma malayanum*, risk factors, diet, control, Philippines, letter

**To the Editor:** In 2002, the Department of Health-Provincial Health Team (DOH-PHT) of Surigao del Norte reported 102 cases of fasciolid-like infections in the municipality of Santa Monica, Siargao Island, the Philippines. The reports were based on characteristic large operculate eggs having been found in routine stool examinations conducted during schistosomiasis surveys. *Fasciola hepatica* infection was the initial diagnosis considered. In 2005, a collaborative team from DOH-PHT, the National Institutes of Health, the University of the Philippines Manila, and the Local Government Unit/Rural Health Unit conducted a field investigation to determine the cumulative prevalence of intestinal helminthic infections in adult patients whose conditions had been previously diagnosed as fasciolid-like infection and to determine the causative species of trematode.

The study group consisted of 70 adult patients from the *barangay* (local government unit of 50–100 families) of Libertad, for whom fasciolid infections had been noted on previous surveys. Researchers confirmed infections and collected adult trematodes for species identification. All patients underwent bowel preparation with bisacodyl (Dulcolax) 10-mg tablets (2 tablets taken immediately after a meal on night before deworming), followed by praziquantel (25 mg/kg in 2 doses 4 h apart), and 30-g magnesium sulfate granules, dissolved in 1 cup of milk, given 1 h after the second dose of praziquantel. Stools were processed by using the Kato-Katz method (*1*) and examined microscopically by medical technologists from the Diagnostic Parasitology Laboratory, College of Public Health, University of the Philippines Manila, for intestinal helminth ova. In addition, a clinical history was taken and a complete physical examination was conducted for each patient volunteer after stool submission. Eating preferences and habits were specifically noted.

The research was approved by the Department of Health Center for Health Development of the Caraga region. Informed consent was obtained before procedures were done and treatment was given to infected patients.

Cumulative prevalence for soil-transmitted helminth infections among the 70 patients was 51.4%. Prevalence according to species was *Trichuris* spp. 42.9%, *Ascaris* spp. 17.1%, and hookworm spp. 1.4%. *Schistosoma japonicum* infection rate was 10%. Stool samples from 8 (11.4%) patients had large (120–130 µm × 80–90 µm), brownish, operculated eggs; 3 had a total of 13 adult flukes. Microscopy showed small leaflike flukes 8–9 mm long and 2.5–3.5 mm wide. After the organisms were processed and stained with aceto-carmine and fast green stains, diagnostic features of *Echinostoma malayanum* (Leiper 1911) were noted. Adult trematodes were within known species size range (5–10 mm × 2.5–3.0 mm) and had elongated bodies and bluntly rounded ends. The ventral sucker (acetabulum) was prominent and larger than the anterior oral sucker. Paired testes were deeply branched and positioned high in the posterior half of the body, extending above the midplane with a single anterior globular ovary. The uterus was entirely anterior to the ovary, and vitellaria (glands) were abundant along both lateral portions of the worm, ending just posterior to the esophagus. The oral sucker had a horseshoe-shaped anterior collar with 43 circumoral spines, which differentiates this species from *E. ilocanum* (49–51 collar spines), another trematode species endemic to the Philippines.

In terms of eating habits, patients reported that fish were commonly eaten raw, after being dipped in a salt and vinegar mixture, locally known as *kinilaw.* Other methods of fish preparation were *tinola* (boiled), *ginataan* (stewed in coconut milk), and *sinugba* (charcoal-grilled). All echinostome-infected patients had a history of having eaten snails, *kuhol* and *kiambu-ay*, prepared raw with coconut milk and lime juice (*kinilaw)*, especially when found in greater abundance during the rainy season.

Human echinostome infection results from ingestion of metacercariae that encyst in secondary intermediate hosts, usually freshwater snails, tadpoles, or fish. *E. malayanum* uses various species of gastropod mollusks for primary and secondary intermediate developmental stages (*2*–*5*). Certain species of fish may also serve as secondary intermediate hosts (*2*). Several mollusks that may serve as primary and secondary intermediate hosts have been identified in the Philippines, including *Lymnaea (Bullastra) cumingiana, Radix quadrasi*, and *Physastra hungerfordiana* for *E. malayanum*, and *Pila luzonica* for *E. ilocanum* (*6*,*7*).

To our knowledge, this is the first report of *E. malayanum* infections in the southern Philippines. Local eating habits are a strong factor in echinostome infections. The general lack of awareness by health staff and the community was a big factor in the poor identification of the disease. Clinical and laboratory staff and healthcare providers need training about echinostome infections and other intestinal foodborne trematode infections. Similar environmental, sanitary, and eating practices in the region suggest that the same parasitoses should be considered to be widespread in the area. Redirecting vital resources of the local health and government units of the Caraga region to the periphery and building local capacity will help empower authorities to provide public health services in rural areas, strengthen public health programs, and further develop public health infrastructure. Moreover, a successful control program against chronic intestinal parasitoses could serve as a paradigm for local health system development of effective control measures for other endemic diseases.
